# Supplementary material for: The Effects of Chimeric Antigen Receptor (CAR) Hinge Domain Post-Translational Modifications on CAR-T Cell Activity
Source: Int J Mol Sci. 2022 Apr 6;23(7):4056. doi: 10.3390/ijms23074056 (PMC8999629; doi:10.3390/ijms23074056)
Supplement: Supplementary file 1 [file ijms-23-04056-s001.zip › ijms-1662886-supplementary.pdf]

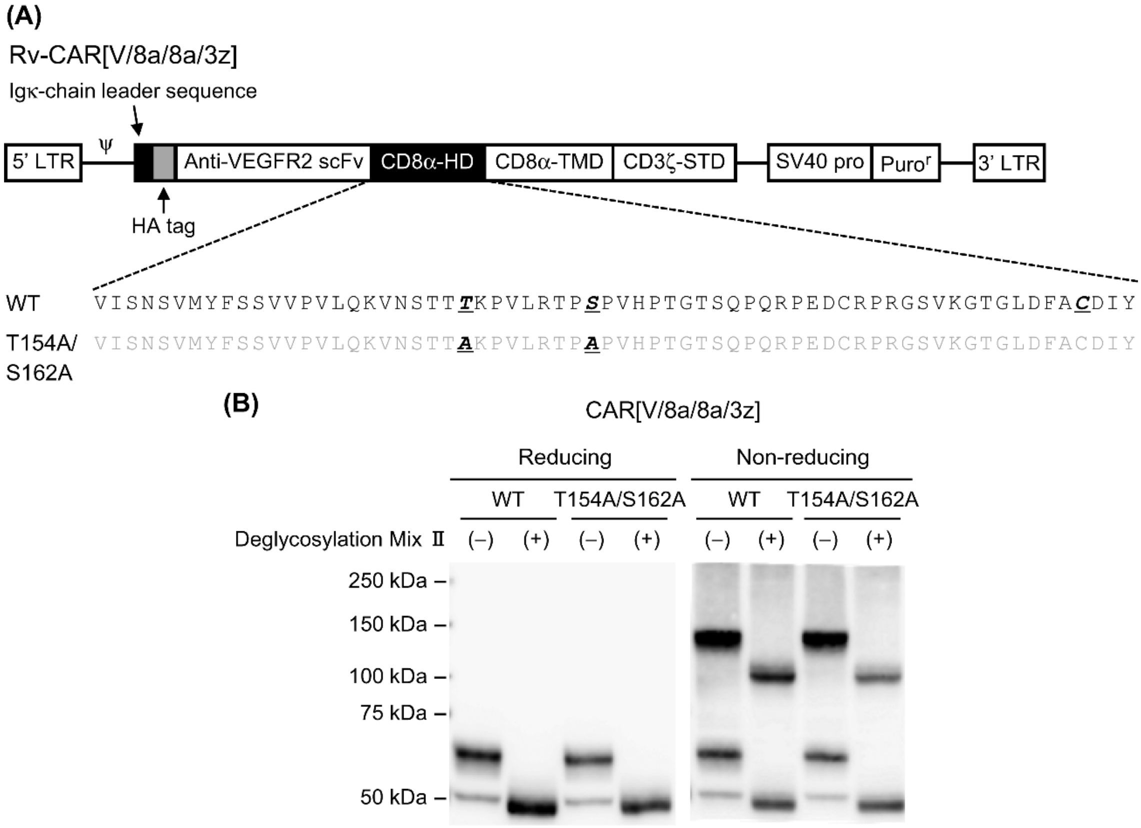

Figure S1. Illustration of Rv construct for the deletion of O-linked glycosylation in HD and analysis of glycosylation of CAR[V/8a/8a/3z]. (A) Illustration of Rv-construct containing the gene encoding a VEGFR2-specific CAR and the amino acid sequence of HD for the deletion of O-linked glycosylation. (B) SDS-PAGE and western blotting analysis showing the expression modality of CARs in whole CAR-T cell lysate on day 0 (24 h after Rv transduction)
